# Supplementary material for: Global and Historical Distribution of Clostridioides difficile in the Human Diet (1981–2019): Systematic Review and Meta-Analysis of 21886 Samples Reveal Sources of Heterogeneity, High-Risk Foods, and Unexpected Higher Prevalence Toward the Tropic
Source: Front Med (Lausanne). 2020 Feb 27;7:9. doi: 10.3389/fmed.2020.00009 (PMC7056907; doi:10.3389/fmed.2020.00009)
Supplement: Supplementary file 1 [file Data_Sheet_1.PDF]

Global and Historical Distribution of *Clostridioides difficile* in the Human Diet (1981-2019): Systematic Review and Meta-Analysis of 21886 Samples Reveal Sources of Heterogeneity, High-Risk Foods, and Unexpected Higher Prevalence Towards the Tropic

Alexander Rodriguez-Palacios DVM PhD[1,2], Kevin Q Mo BSc[3,4], Bhavan U. Shah MD MPH[5,6], Joan Msuya MSc[7], Nina Bijedic PhD[8,9], Abhishek Deshpande MD PhD[6], Sanja Ilic PhD[3]

[1]Division of Gastroenterology and Liver Disease, and [2]Digestive Health Research Institute, Case Western Reserve University School of Medicine, Cleveland, OH 44106, USA
[3]Department of Human Sciences, Human Nutrition, College of Education and Human Ecology, The Ohio State University, Columbus, OH 43210, USA
[4]Northeast Ohio University of Medicine, Rootstown, OH 44272, USA
[5]Informatics and Assessment Division, Lorain County General Health District, Elyria, OH 44035, USA
[6]Department of Neurology, Weill Cornell Medicine, Cornell University, New York, NY 10065, USA
[7]Department of Health and Nutrition, World Vision, Arusha, Tanzania
[8]Department of Applied Mathematics and Formal Methods, Information Technologies, University Dzemal Bijedic, Mostar 88000, Bosnia and Herzegovina.
[9]Department of Mathematics, University of North Carolina, Charlotte, NC 28262, USA
[10]Medicine Institute Center for Value-Based Care Research, Cleveland Clinic, Cleveland, OH 44106 USA

Correspondence:
axr503@case.edu (AR-P)
ilic2@osu.edu (SI)

Supplementary Materials

Content

1. **Supplementary Tables 1-7**
2. **Supplementary Figures 1-7**
3. **Supplementary Materials and Methods**.
    a. Search Algorithm and Inclusion/Exclusion Criteria Definitions
    b. Supplementary Statistics
4. **Supplementary References**.

Supplementary Table 1. Level 1 - Relevance Screening Tool Checklist for Title and Abstract

Question	Options	Definitions/additional notes
RefID	Will be retrieved automatically from the software	
1. Does the abstract investigate **C. difficile in any type of animal origin food?**	☐ Yes<br>☐ No (excluded, submit form)<br>☐ Not clear	**At this initial stage of the study: *Animal origin food* is any food for human consumption produced from or by** an animal of any sort, including insects, and for the purposes of this study, relate to animals found in North America.<br>***Pathogenic bacteria*** are any bacteria that cause infectious diseases.
ONLY PROCEED IF YOU ANSWERED YES TO Q1		
**2.** Is the main focus of the study **prevalence of C. difficile in the investigated food?**	☐ Yes<br>☐ No (excluded, submit form)<br>☐ Not clear	***Prevalence***, the total number of positives in a sample population at a specific time (numerator, denominator).
**ONLY PROCEED IF YOU ANSWERED YES TO Q2 OTHERWISE SUBMIT FORM**		
3. Is this abstract primary research in any language?	☐ Yes, primary research<br>☐ No, systematic review /meta-analyses<br>☐ No, literature review or commentary<br>☐ No, other, specify: _________	***Primary research***<br>Primary research represents a study where the authors collected and analyzed their own data – may use quantitative or qualitative methods or both to investigate the research question and report original results.<br>***Systematic reviews and meta-analyses***<br>Any systematic review or meta-analysis that identifies, appraises and summarises, included primary research relevant to the research question<br>***Review/commentary***<br>A comprehensive or brief narrative review or commentary (from peer-reviewed articles to lay magazine or newspaper articles or briefs) describing and/or discussing the issue of C. difficile in any animal origin food
3. Food of animal origin investigated (please check all that apply)	☐ *Meat (not specified)*<br>☐ *Beef*<br>☐ Ground/minced *beef*<br>☐ *Poultry (chicken)*<br>☐ *Turkey*<br>☐ *Pork*<br>☐ Other, please specify___	

Reviewer Decision:
If the reviewer selects "No to questions 1- 3" the article will be excluded. All included abstracts will be procured as full articles and will be further evaluated using the data characterisation and utility form (level 2).

Supplementary Table 2. Level 2 - Quality Assessment and Data Extraction Tool for screening of selected Full-Text Articles

Cdiff Foods SR QA/DE tool for prevalence articles		
1. Does this study contain data relevant to prevalence of C. difficile in foods?<br>Rel_data	a. Yes 1<br>b. No 0<br><br>If 'no' please stop and contact Sanja Ilic<br>If "yes" please continue with the spread sheet	a. Study contains relevant data on prevalence of C. difficile in food, with clear numerator and denominator presented e.g. 3 of 100 meat samples tested positive.<br>b. Study does not contain relevant data with clear numerator and denominator e.g. only reports characterization of previously collected isolates, with no clear denominator.
**Publication details - Location**		
If the study contains two sets of data for the same sample due to different culture media or a different time point, please use 'a' for first media, 'b' for second media, etc.)		
2. What is this study ID#<br>RefID	a. text	ID# Please use PMID from pubmed if not available use the reference number from WebEndNote
3. Data Extraction user User	a. text	Name of the person who is extracting the data
4. Verification Ver	a. text	Name of the person who is verifying the data
5. First Author Author	a. text<br>b. unknown 0	Please use this format:<br>Author Last Name, Initials
6. What year was this study published? Pub_year	a. text<br>b. unknown 0	Publication year: full year of the manuscript publication, e.g. 2012
7. Where was the study conducted?	a. Africa 1<br>b. Asia 2<br>c. Australia/New Zealand 3<br>d. Europe 4<br>e. North America 5<br>f. Latin America/Caribbean 6<br>g. Other:________text<br>h. Not reported 0	
8. Specify country where the study was published Pub_country	a. text<br>b. unknown 0	Publication country: the country that the study was published usually available with affiliations
9. What is the name of the journal? Journal	a. text<br>b. unknown 0	Journal name: full name of the journal in which the study was published
10. What institution(s) funded the study? Funding	a. text<br>b. unknown 0	Name of institution(s) listed in acknowledgements of the manuscript that funded the study
**Study Design**		
11. What is the study design, as identified by the reviewer?<br><br>Stdy_dsgn	a. Prevalence survey 1<br>b. Longitudinal prevalence 2<br>c. Surveillance 3<br>d. Challenge trial 4<br>e. Controlled trial 5<br>f. Quasi-experiment 6<br>g. Cohort study 7<br>h. Case-control study 8<br>i. Cross-sectional study 9<br>j. Case report or series 10<br>k. Other (please specify) text	**Prevalence survey:** A study that measures, and may describe (e.g. concentration), the degree of contamination of a food by C. difficile at a particular point in time.<br>**Longitudinal prevalence**: A study that measures, and may describe (e.g. concentration), the degree of contamination of a food by C. difficile over two or more time intervals. Samples may either be at the level of the location (e.g., supermarkets; processing facilities) or the product (e.g. a set of 10 dry-fermented sausages sampled three times over several weeks)<br>**Surveillance:** A system that continuously gathers, analyzes, and interprets data about diseases (or contamination of certain foods) and disseminates conclusions of the analyses to relevant organizations in a timely manner.<br>**Challenge trial:** An experiment where foods are artificially challenged or exposed to C. difficile for the purpose of characterizing C. diff in the food<br>**Controlled trial:** An experiment where an intervention is applied to contaminated foods or relevant environment(s) (e.g. processing facilities) for the purpose of reducing or eliminating C. difficile.<br>**Quasi-experiment:** An experiment where an intervention is applied to contaminated foods or relevant environment(s) (e.g. processing facilities) in a non-randomized fashion for the purpose of reducing or elimination C. difficile (e.g. Before and after trial)

		**Cohort study**: An observational study where multiple measurements of a sample population of foods or affected persons or relevant environment(s) (e.g. processing facilities) are obtained over two or more time periods to identify risk factors for contamination with C. difficile. Can be either retrospective or prospective.<br>**Case-control study**: An observational study where contaminated foods or affected persons or relevant environments (e.g. processing facilities) are matched with non-contaminated foods, affected persons or relevant environments, respectively, to identify risk factors for contamination with C. difficile or its vehicles.<br>**Cross-sectional study**: An observational study where foods, or relevant environment(s) (e.g. processing facilities) are sampled for the purpose of identifying or characterizing the degree of contamination, as well as potential risk factors for contamination with C. difficile.<br>**Case report or series**: A descriptive study that tracks affected persons with a foodborne disease for the purpose of identifying the etiological agent (C. difficile), vehicle of transmission (foods) and source/point of contamination. Includes preliminary assessment that includes qualitative/quantitative questionnaires of affected persons, collection of clinical specimens, collection of food and environmental samples, but does not include further epidemiological investigation (e.g. case-controls).
12. Is raw or unadjusted data provided?<br>QA_rawdata	a. Yes 1<br>b. No, specify reason________	**Yes:** For prevalence data, the following data must be reported<br>• Numerator and denominator, or<br>• Proportion + EITHER numerator or denominator<br><br>**For measures of association/effect:**<br>• OR/RR/IR/RD reported and its measure of variability (SE, SD, CI) or P-value is provided<br><br>**For continuous measures:**<br>• Mean value, sample size, and SD<br>• Mean value and SE/CIs<br><br>**Examples of no:**<br>a. Graphical data only<br>b. No reporting of raw results<br>c. Just median<br>d. Only p-value<br>e. Only denominator<br>f. Only numerator.
**Foods**		
13. What specific food product was measured?<br>Food_name	a. Text. . ………………………..<br>0. Not described by author	Food name: for instance lettuce, beef, poultry, vegetables, etc.
14. What food category was measured? Food_ cat	a. Ready to Eat<br>b. Raw<br>c. Processed<br>d. Other (please specify)………<br>0. Not reported 0	**RTE** are foods that will be consumed without any further preparation of processing<br>**Raw** are previously unprocessed foods that are commonly consumed cooked<br>**Processed** foods that will that are usually consumed as cooked.<br>Other: if category is not specified above, indicate here and provide description
15. In what country(s) did food sample collection take place?<br>Country	a. TEXT<br>b. Not described 0	Name of the country or countries that the samples were collected in. This is not the origin of the food
16. In what city did food sample collection take place?<br>City	a. TEXT<br>b. Not described 0	Name the city or cities that the samples were collected in. This is not the origin of the food
17. What kind of area does the city where the sample collection took place belong to?<br>Urban/rurall	a. Urbanized areas 1<br>b. Urban clusters 2<br>c. Rural 3<br>d. Unknown 0	**Urban areas** (UAs) of 50,000 or more people<br>**Urban Clusters** (UCs) of at least 2,500 and less than 50,000 people.

		**Rural** encompasses all population, housing, and territory not included within an urban area. Whatever is not urban is considered rural.
18. What is the origin of food? Food_orgn	a. Text<br>b. Not described 0	Name of the country were the food was originally produced. This is the origin of food.
19. Where in the food production chain was the sampling conducted? Chain	a. Farm 1<br>b. Commercial processing plant 2<br>c. Retail 3<br>d. RTE 4<br>e. Import-Export 5<br>f. Research lab 6<br>g. Not reported 0	**Farm**: Location of commercial production/harvesting of foods (e.g., farm, orchard, etc.). (i.e., products that will later be sold to consumers).<br>**Commercial processing plant**: Location of processing and/or packaging of foods (e.g., sausage processing facility, facilities to process fresh spices and herbs into packed products).<br>**Retail**: Any location where consumers can purchase foods (e.g., local grocery stores, supermarkets, farmer's markets, butcher's shops).<br>**Ready-to-eat**: Locations that serve/offer foods and products containing these foods that can be immediately consumed. (e.g., restaurants, delicatessens, cafeterias, buffets, etc.)<br>**Import/Export**: Foods are sampled immediately before they leave the country of production or immediately after they enter the country of sale.<br>**Research/laboratory facility**: Articles that report on a study sampling products in a laboratory setting.
20. When was the study conducted? Time_period	a. Mm/yyyy to mm/yyyy<br>b. Not reported 0	
21. Was the sample representative of the larger/target population QA_rep	a. Yes<br>b. No	**Yes:** At least some information provided to reflect the representativeness of the sampled population to the target population.<br>**No**: No explanation of external validity of results was given and thus, the sample may not be representative.
22. How were sampling units (retail stores, restaurants, farms) selected in this study? (E.g. random ..stores) QA_units	a. Random…….1<br>c. Reported random…….2<br>d. Systematic………3<br>e. Convenience……..4<br>99. Not applicable	**Random**: Computer or random numbers table, a priori, stratified random sample, cluster random sample.<br>**Reported random:** Author indicates random, but randomization is not explained.<br>**Systematic:** Taking n samples at interval of x.<br>**Convenience:** Stores selected in the vicinity, or it was not described in the paper.<br>**99:** Units were not selected to participate in this study, or this row does not pertain.
23. How were individual samples selected in this study? QA_samples	a. Random…….1<br>b. Reported random…….2<br>c. Systematic………3<br>d. Convenience……..4<br>99. Not applicable	**Random:** Computer or random numbers table, a priori, stratified random sample, cluster random sample.<br>**Reported random:** Author indicates random, but randomization is not explained.<br>**Systematic:** Taking n samples at interval of x.<br>**Convenience:** it was not described in the paper.
24. Was the sample size (number of samples) justified? QA_ss	a. Yes 1<br>b. No 0	**Yes**: Use of sample-size formulas, based on desired power or precision and estimate of expected variability to detect differences, or the author justified the sample population is the census population/maximum accessible.<br>**No:** No details regarding how sample size was determined or the author describes informal guesses of sample size.
25. Were laboratory methods pertaining to this specific row of data used to determine the outcome described sufficiently to allow replication of the study? QA_labmeth	a. Yes 1<br>b. Reference paper 2<br>c. No 0	**Yes:** Methods are reported in sufficient detail to allow for replication.<br>*(Culture media, enrichment, pre-enrichment, PCR- PCR type, primer name, length, target sequence)*<br>Referenced paper: Methods are referenced in another paper.<br>**No:** Not sufficiently reported to be able to reproduce the study without contacting the author.
26. Was the statistical analysis described adequately so it can be reproduced? QA_statanalysis	a. Yes 1<br>b. Reference paper 2<br>c. No 0<br>d. Statistical analysis not done 99	**Yes:** The methods were reported in sufficient detail to understand the statistical approach and reasoning.<br>**Referenced paper:** Methods referenced in another paper.<br>**No:** Methods and adjustments are not clear or some details are missing.<br>**Statistical analysis not done**: Statistics not reported.<br>.

**Laboratory methods<br>Tool created based on C difficile reporting order from Rodriguez-Palacios 2016**		
**Overall culture approach<br>(read the whole methods section first then answer section below, and copy paste section to the right)**		
Which option resembles the culture method best?	a. Sample > **direct plating 1**<br>**b.** Sample > heat/ethanol_selection > **direct plating 2**<br>**c.** Sample > heat/ethanol_selection > preenrichment > plating 3<br>d. Sample > heat/ethanol_selection > preenrichment > selection_again > plating 4<br>e. Sample > preenrichment > plating 5<br>f. Sample > preenrichment > heat/ethanol_selection > plating n6<br>g. other 99	
**SAMPLE handling and storage**<br>(how the sample was stored after collection and for how long. Important: prolonged storage lowers recovery rate)		
Were samples/analysed using blinding analysis?	a. Yes 1<br>b. No 0	Yes- blinded analysis was reported by authors<br>No - blinded analysis was not performed or not reported by authors<br>Example: Selected samples were recoded for blinding purposes , YES should be chosen
At what temperature were the samples stored/transported prior to processing?	a. Room temperature 1<br>b. Refrigerated 2<br>c. Frozen_-20oC 3<br>d. Frozen_-80oC 4<br>e. Other 99	Example: samples were stored at –80°C within 24 hours of collection.
How long were the samples stored following collection and before the sample processing?	a. Enter days<br>b. Not reported	Storage length can vary. The early studies enriched samples for 7-14 days. But shorter periods have been reported. Enter length in days. If 12 hours, enter 0.5 days. If 18 hours, enter 0.75 days
What amount of the sample was used for enrichment or culture?	a. Enter grams/sample<br>b. Not reported	Specify the amount and units (grams)
Was the sample processed/cultured in replicates?	a. Single replicate<br>b. Duplicate<br>c. Triplicate<br>d. Other<br>e. Not reported	Samples process in duplicate are more likely to yield a positive value.
**Enrichment incubation**<br>(sample is FIRST placed on liquid medium to allow C difficile to outgrow/compete other bacteria)		
Were pre-treated to eliminate other bacterial cells, selecting for spores and thus facilitate the enrichment/isolation of C. difficile?	a. Ethanol, specify 1<br>b. Heat, specify 2 text<br>c. No 0<br>d. Other, specify text	**Ethanol**: sample treated with ethanol prior to enrichment<br>**Heat**: sample heated at 80oC for certain time, specify temperature and time<br>**No:** sample placed directly to enrichment<br>**Other**, specify: specify treatment and parameters<br><br>**Example**: Briefly, example: Briefly, t and parameters<br>Ethanol 1
27. What enrichment media was used in this study?	a. No enrichment 0<br>b. Brain Heart Infusion (BHI) 1<br>c. Clostridium difficile agar base BROTH 2<br>d. Other broth, specify text	**Enrichment media** used means that the sample was incubated in a SOLUTION or BROTH to allow the spores of *C. difficile* to grow and then become spores again to enhance the number, PRIOR TO plating them on a solid agar for identification of colonies.<br>**Clostridium difficile agar base BROTH**: Initial studies were conducted using a broth made in house with the reagents used for a commercial C. difficile agar without the agar.<br>**Enrichment not used** – this is the case when samples were processed/plated DIRECTLY on agar culture media to allow the formation of colonies for testing and recognition based on morphology.<br><br>**Example:** Sediment was recovered after centrifugation at 3,800 □□*g* for 10 minutes and resuspended in 5 mL of cycloserine-cefoxitin fructose broth (*C. difficile* agar and *C difficile* supplement SR0096; Oxoid, Columbia, MD, USA) that was incubated anaerobically at 37°C for 7 days.<br>*Clostridium difficile* agar base BROTH should be selected

28. Was antibiotic used during enrichment?	a. No antibiotics used 0<br>b. Cycloserine/cefoxitin (CCF supplement) 1<br>c. Moxalactem/Norfloxacin supplement (CDMN) 2<br>d. Other antibiotic, specify text	Most common antibiotic combinations come from these two commercial products:<br>-CLOSTRIDIUM DIFFICILE SELECTIVE SUPPLEMENT Cycloserine/cefoxitin (CCF supplement)<br>-CLOSTRIDIUM DIFFICILE MOXALACTAM NORFLOXACIN (CDMN) SELECTIVE SUPPLEMENT. Moxalactem/Norfloxacin supplement (CDMN)<br>Report other if used.<br><br>**Example:** The sediment was recovered after centrifugation at 3,800 □□*g* for 10 minutes and resuspended in 5 mL of cycloserine-cefoxitin fructose broth (*C. difficile* agar and *C difficile* supplement SR0096; Oxoid, Columbia, MD, USA) that was incubated anaerobically at 37°C for 7 days.<br>Cycloserine/cefoxitin (CCF supplement) should be reported
28. What incubation temperature was the enrichment performed?	a. Specify temperature in ºC text<br>b. Not reported 0	Enrichment is usually done at 37ºC. Enter the number in ºC
29. What was the incubation period?	a. Specify number of days.<br>b. Not reported 0	Enrichment length can vary. The early studies enriched samples for 7-14 days. But shorter periods have been reported. Enter length in days. If 12 hours, enter 0.5 days. If 18 hours, enter 0.75 days
**Plating: culture on agar**<br>(plating of sample, or of the enrichment broth sediment onto solid agar surface)		
30. What culture media base was used in this study?	a. Tryptic Soy Agar 5<br>b. Brain Heart Infusion (BHI) Agar 1<br>c. OXOID Clostridium difficile agar 2<br>d. BRAZIER Clostridium difficile agar 3<br>e. Chromeagar C. difficile 4<br>f. Other agar, specify<br>g. Not reported 0	There are only a few companies that make C. difficile agar, for example OXOID. Except of BHI, we will get the ingredients of the culture media from the company labels. If the company does not match, please select Other and specify the name and company
30. What type of blood was added to the media base used in this study?	a. No blood added 0<br>b. Horse laked blood<br>c. Sheep defirbinated blood<br>d. Bovine defibrinated blood<br>e. Other, specify	Blood is added to media for enrichment, if can be derived from a number of animal species. The blood can be pre-treated prior to addition to prevent coagulation. Laked means the red blood cells were lysed. Defibrinated signifies that blood fibrin was removed keeping the red blood cells intact which is ideal for assessment of hemolysis. C. diff is not hemolytic. Several combinations of animal species and pre-treatment are possible and most commonly used are these listed. If other was used please specify the animal species and the treatment.
31. What antibiotic was added to the media base used in this study?	a. No antibiotics used 0<br>b. Cycloserine/cefoxitin (CCF supplement) 1<br>c. Moxalactem/Norfloxacin supplement (CDMN) 2<br>d. Other antibiotic, specify text	Most common antibiotic combinations come from these two commercial products:<br>-CLOSTRIDIUM DIFFICILE SELECTIVE SUPPLEMENT Cycloserine/cefoxitin (CCF supplement)<br>-CLOSTRIDIUM DIFFICILE MOXALACTAM NORFLOXACIN (CDMN) SELECTIVE SUPPLEMENT. Moxalactem/Norfloxacin supplement (CDMN)<br>Specify other if used.
28. What incubation temperature was the enrichment performed?	a. Specify temperature in ºC text<br>b. Not reported 0	Enrichment is usually done at 37ºC. Enter number in ºC
29. What was the incubation period?	a. Specify number of days.<br>b. Not reported 0	Enrichment length can vary. The early studies enriched samples for 7-14 days. But shorter periods have been reported. Enter length in days. If 12 hours, enter 0.5 days. If 18 hours, enter 0.75 days
What positive controls were used?	a. Yes, ATCC or NCTC<br>b. Yes, other<br>c. No positive control used	**ATCC** American Type Culture Collection<br>**NCTC** National Collection of Type Cultures<br>Yes, other is to be chosen when an internal laboratory collection strains were used or other collections.
What negative controls were used?	a. Text<br>b. No negative control used	Specify any negative control species, or water used as negative control

**Biochemical and molecular characterization of isolates**		
How many isolates were selected per sample for biochemical and molecular confirmation?	a. Text<br>b. Not reported	
How many isolates were selected per sample for biochemical and molecular confirmation?	a. Text<br>b. Not reportes	
32. What method was used to confirm the C. difficile isolates was?	a. L-proline amminopeptidase activity<br>b. Gram-stain<br>c. 16S sequence<br>d. tpi gene<br>e. gldh gene<br>f. PCR testing of C. difficile toxin genes<br>g. Other<br>h. Not reported	C. difficile isolates are confirmed using various biochemical and molecular typing techniques. Please check all that apply.<br><br>Example
What genetic methods where used to compare the isolated strains to one another (genotyping)? List all that apply	a. PCR ribotyping<br>b. Toxinotyping<br>c. RFLP<br>d. PFGE<br>e. REA<br>f. MLST<br>g. MLVA<br>h. Whole genome sequencing<br>i. Other	C. diff typing is based on DNA extracted from isolates. The DNA can be amplified, cut using restriction enzymes or amplified and sequenced. The most common methods are standard and the typical abbreviations are following:<br>PCR ribotyping: Polymerase chain reaction amplification of intergenic region 16s-23s.<br>RFLP: restricted fragment length polymorphism<br>Toxinotyping: is an RFLP restricted to the toxins A and B regions. Nomenclature assigned follows roman numbers 0, I, II, III. IV, V…XXV..<br>PFGE: Pulse field gel electrophoresis. Nomecnlaluter starts with NAP1…(north American pulsotype 1)<br>REA: restriction enzyme analysis<br>MLST: Multilocus sequence typing<br>MLVA: Multilocus variable number tandem repeat analysis<br>Whole genome sequencing<br><br>**Example: PCR Ribotyping** PCR ribotyping analyses were performed as previously described (*22*). DNA was amplified by using a thermal cycler (Touchgene Gradient, Techne Inc., Burlington, NJ, USA). Ribotype patterns were compared visually with *C. difficile* PCR ribotypes from humans and other animals from the provinces of Ontario, Quebec, and Manitoba, Canada. The first isolate identified for each PCR ribotype was submitted to the Anaerobe Reference Laboratory, University Hospital of Wales, Cardiff, United Kingdom, for comparison (*23*).
If PCR ribotyping was used, there is evidence there was an international reference set of isolates for comparison and for positive controls?	a. Yes<br>b. No	*PCR ribotyping (the oldest based on U of Wales, Cardiff, UK) and MLST are among the best recognized international typing nomenclature systems.*<br><br>*PCR ribotyping* International nomenclature uses a three digit number with no letters PCR-ribotype 027, or PCR-ribotype 078. If letters are part of the nomenclature it implies that the typing was based on local designations without international referent isolates. For instance PCR ribotype A, or S12, incodate the designations were created for local comparisons initially.
Were the isolates molecularly (PCR) tested for the presence of the following toxin genes?	a. toxins tcdA or tcdB or both 1<br>b. binary cdtA or cdtB or both<br>a. all tcdA&B; cdtA&B,<br>b. No 0	*Clostridium difficile* may be toxigenic or nontoxigenic depending on the presence or absence of at least one of the three toxin genes: A (tcdA), B (tcdB) or Binary toxin (cdtA&B). Ideally the conformation of such genes has to done by detecting the gene using PCR. This question is about the use of PCR to confirm those toxin genes, and the toxin regulator tcdC (which may determines how much toxins are produced).<br><br>**Example**: Amplification of nonrepeating and repeating sequences of the tcdA gene and the nonrepeating

		sequences of the tcdB gene was performed as previously described (24). Identification of tcdC and cdtB genes was based on previous protocols (11,24,25). Reference strains were included.
Were the isolates PCR tested for the presence/mutations of the toxin gene regulator tcdC?	a. Yes<br>b. no	The toxin regulator tcdC may have mutations and determine how much toxins A and B are produced in vitro and possibly in vivo by C. diffiicle. In the example above, tcdC was tested.<br><br>The answer is Yes
Were the isolates tested for toxin production?	a. Yes, using cell culture assay with confirmation by toxin neutralization 1<br>b. Yes, using cell culture assay without toxin neutralization 2<br>c. Yes, using ELISA 3<br>d. Both, cell line and ELISA 4<br>e. No / not reported 0	To determine if *Clostridium difficile* may be toxigenic or nontoxigenic it is also necessary to confirm the actual production of the toxin molecules (protein) by testing the virulence activity of C.difficile culture supernatants on monolayers of cell lines, or by using ELISA against the toxin proteins. On cell lines, toxins produce a cytopathic effect (makes the cells round and die). With ELISA, the assay only confirms there are proteins that antigenically interact with the specific antibodies present in the ELISA kit.
**Antimicrobial resistance**		
What antimicrobials were tested in this study?	a. Text<br>b. No antimicrobials tested 0	
What kind of antimicrobial resistance test was used to test the isolates	a. No AMR tested 0<br>b. E-test 1<br>c. Disk diffusion 2<br>d. Microbroth dilution 3<br>e. Agar dilution 4<br>f. Other, specify text	
**Outcome measurement**		
40. Sample Wt./Vol.<br>Sple_wgt	a. Text.<br>b. Not reported 0	The measure of sample weight, volume or area. Please indicate units as reported.
41. Number of samples per pool<br>Pool_sple	a. Text.<br>0. Not reported<br>99. Not applicable	Please indicate the number of samples pooled together prior to laboratory analysis.
42. Pool weight<br>Pool_wgt	a. Text.<br>0. Not reported<br>99. Not applicable	The sum of the pooled sample weight. Please indicate units as reported (eg. grams)
43. Unit of observation for this specific row of data.<br>**Obs_unit**	a. Text<br>0. Not reported	Level at which this row of data was gathered- individual food or food category
**44. Other specific descriptors of the population to which this specific row of data pertains**<br>Prev_oth	a. Text<br>99. Not applicable	For example, ‘hospital restaurants’, etc
45. What toxigenic genotypes were targeted or recovered?	a. Text<br>PCR-ribotyipe 001<br>PCR-ribotyipe 027<br>PCR-ribotyipe 078<br>PCR-ribotyipe 017<br>PCR-ribotyipe 077<br>PCR-ribotyipe 014<br>PCR-ribotyipe 126<br>b. Other enter<br>c not specified 0<br>99. Not applicable	Among the most virulent and epidemic PCR ribotypes, the ones listed are known to have particular relevance in the epidemiology and severity of C. diff infections in humans.
45. Were any non-toxigenic isolates recovered inthis study?	a. yes<br>b. no.<br>c. not specified 0<br>99. Not applicable	Non-toxigenic isolates do not cause disease but they have been considered to have a probiotic preventive role in human disease.

DICHOTOMOUS RAW DATA (prevalence)		
46. Number of samples positive.<br><br>Sple_pos	a. Text.<br>0. Not reported	Refers to number of samples collected, testing positive.
47. Prevalence<br>Prev	a. Text.<br>0. Not reported	Number of positive units divided by the total number of units.
48. Prevalence 95% CI low<br>Prev_ci_lo	a. Text.<br>0. Not reported	Lower 95% confidence interval.
49. Prevalence 95% CI high<br>Prev_ci_hi	a. Text.<br>0. Not reported	Upper 95% confidence interval.
50. Prevalence SE/SD<br>Prev_se_sd	a. Text.<br>0. Not reported	Standard error or standard deviation of prevalence estimate<br>(Please indicate which).
**CONTINUOUS RAW DATA (concentration)**		
51. Mean, please specify the name of this value.<br>OD_mean	a. Text.<br>0. Not reported	If average mean outcome is reported please indicate type of outcome and purpose e.g. mean OD or titres etc.
52. 95% CI low<br>OD_ci_lo	a. Text.<br>0. Not reported	Lower 95% confidence interval.
53. 95% CI high<br>OD_ci_hi	a. Text.<br>0. Not reported	Upper 95% confidence interval.
54. SE/SD<br>OD_se_sd	a. Text.<br>0. Not reported	Standard error or standard deviation of continuous estimates<br>(Please indicate which).

Supplementary Table 3 – Food Categories Defined and Designated by Reviewers' Consensus for the Present Meta-analysis on *C. difficile* in the Human Diet

Food Categories	Freq.	Percent	Cum.
Milk	5	2.17	2.17
baby_formula	2	0.87	3.04
**beef**	48	20.87	23.91
camel	1	0.43	24.35
grain	3	1.30	25.65
honey	2	0.87	26.52
lamb	9	3.91	30.43
**leafyg**	16	6.96	37.39
live_poultry	1	0.43	37.83
meat_product	3	1.30	39.13
mixed_meal	18	7.83	46.96
mixed_meat	12	5.22	52.17
mixed_vegfruits	3	1.30	53.48
ostrich	1	0.43	53.91
**pork**	30	13.04	66.96
poultry	34	14.78	81.74
root/soil vegetables	15	6.52	88.26
**seafood**	24	10.00	98.26
seasoning	1	0.43	98.70
water	3	1.30	100.00

Supplementary Table 4 – Arithmetic Study Average of *C. difficile* in Food Items Across Studies.

study_aver	Freq.	Percent	Cum.
0	11	14.10	14.10
.1	1	1.28	15.38
.3	1	1.28	16.67
.6	1	1.28	17.95
.66	2	2.56	20.51
.9	1	1.28	21.79
1	1	1.28	23.08
1.06	1	1.28	24.36
1.2	1	1.28	25.64
1.4	1	1.28	26.92
1.5	2	2.56	29.49
1.8	2	2.56	32.05
2	3	3.85	35.90
2.1	2	2.56	38.46
2.31	1	1.28	39.74
2.6	1	1.28	41.03
2.8	1	1.28	42.31
3	2	2.56	44.87
3.1	1	1.28	46.15
3.2	1	1.28	47.44
3.3	1	1.28	48.72
3.5	1	1.28	50.00
3.7	1	1.28	51.28
4.5	1	1.28	52.56
4.8	1	1.28	53.85
4.9	1	1.28	55.13
5.7	2	2.56	57.69
6.3	2	2.56	60.26
6.5	1	1.28	61.54
6.9	1	1.28	62.82
7.3	1	1.28	64.10
7.8	1	1.28	65.38
10.4	1	1.28	66.67
10.5	1	1.28	67.95
10.6	1	1.28	69.23
12.2	2	2.56	71.79
13.3	1	1.28	73.08
13.8	1	1.28	74.36
14	1	1.28	75.64
15.115	1	1.28	76.92
15.8	1	1.28	78.21
16	1	1.28	79.49
17.5	1	1.28	80.77
17.8	1	1.28	82.05
19.57	1	1.28	83.33
20.9	1	1.28	84.62
21.9	1	1.28	85.90
25.2	1	1.28	87.18
27.7	1	1.28	88.46
29	1	1.28	89.74
29.1	1	1.28	91.03
30	1	1.28	92.31
33.7	1	1.28	93.59
38.2	1	1.28	94.87
47.4	1	1.28	96.15
52.9	1	1.28	97.44
61.1	1	1.28	98.72
100	1	1.28	100.00
Total	79	100.00	

Supplementary Table 5 – Distribution of Catalogued Culture Strategies Identified in this Study: Meta-analysis on *C. difficile* in the Human Diet

Overall Culture Strategy Codes	Frequency	Percent	Cumulative %
0 (not deducible/well described)	8	3.48	3.48
1	6	2.61	6.09
Both 1, and 5	2	0.87	6.96
2	6	2.61	9.57
Both 2, and 6	3	1.30	10.87
3	4	1.74	12.61
Either 3, 5, or 6	4	1.74	14.35
5	27	11.74	26.09
6	165	70.87	96.96
6, and other	3	1.30	98.26
99 (not reported)	4	1.74	100.00
Total	232	100.00	

232 Food Sample Sets were Tested (in 79 studies) based on Six Possible Microbiological Culture Approaches. See definition of culture approach codes 0 through 6, and statistics in **Figure 2A-C**. Strategies defined by consensus between senior microbiologists (ARP and SI).

Supplementary Table 6 – Most studies do not use positive controls to ensure culture method is strictly anaerobic

51_Culture type of positive controls	Freq.	Percent	Cum.
0 (No positive controls used)	58	74.36	74.36
1yes (not described)	2	2.56	76.92
ATCC	2	1.28	78.21
ATCC 43255	1	1.28	79.49
C. difficile ATCC 43255	1	1.28	80.77
C. difficile ATCC 9689	1	1.28	82.05
C. perfringens (NCTC 8237)	1	1.28	83.33
Cdiff ATTC 9689, NCTC 11223, and CCU..	1	1.28	84.62
Cdiff 1035	1	1.28	85.90
Cdiff ATCC 43255	1	1.28	87.18
Cdiff ATCC 700057 Bacteroides fragil..	1	1.28	88.46
Cdiff ATCC 9689	1	1.28	89.74
Cdiff ATCC 9689 and Cperfringens ATCC	1	1.28	91.03
Cdiff ATCC BAA 1870	1	1.28	92.31
Cdiff CD41	1	1.28	93.59
Cdiff cultures	1	1.28	94.87
Cdiff from cattle, retail meats, hum..	1	1.28	96.15
Cdiff ribotype 027	1	1.28	97.44
RT089 RT126 RT127 RT017 RT002 RT 017	1	1.28	98.72
notspecified	1	1.28	100.00
Total	79	100.00	

Supplementary Table 7 – Meta-regression coefficients from a model designed to test the ranking significance among reported prevalences of *C. difficile* among 20 food type categories controlling for culture methods, continent, sample size, and latitude.

```
Meta-regression                                    Number of obs  =     226
REML estimate of between-study variance            tau2           =       0
% residual variation due to heterogeneity          I-squared_res  =   0.00%
Proportion of between-study variance explained     Adj R-squared  = 100.00%
Joint test for all covariates                      Model F(35,190)=    2.52
With Knapp-Hartung modification                    Prob > F       =  0.0000
```

_es_allprev	exp(b)	Std. Err.	t	P>|t|	[95% Conf.	Interval]
_totalnumberofsamples	.9999315	.0000334	-2.05	0.042	.9998655	.9999975
_Ifoodcateg_1	.885635	.0534036	-2.01	0.045	.7863184	.9974959
_Ifoodcateg_2	.9485071	.1133983	-0.44	0.659	.749244	1.200765
_Ifoodcateg_3	.9236978	.0333341	-2.20	0.029	.8602311	.991847
_Ifoodcateg_4	.8809176	.0852386	-1.31	0.192	.7278537	1.06617
_Ifoodcateg_5	.8286793	.0902425	-1.73	0.086	.6684934	1.027249
_Ifoodcateg_6	.8168584	.0860637	-1.92	0.056	.6635746	1.00555
_Ifoodcateg_7	.8736569	.0445184	-2.65	0.009	.7901121	.9660356
_Ifoodcateg_9	.9689598	.1269103	-0.24	0.810	.7483492	1.254606
_Ifoodcateg_10	.9108092	.0797654	-1.07	0.287	.7663097	1.082556
_Ifoodcateg_11	.8677732	.0322311	-3.82	0.000	.8064695	.9337369
_Ifoodcateg_12	.8680167	.0422247	-2.91	0.004	.7885985	.955433
_Ifoodcateg_13	.824799	.0888304	-1.79	0.075	.6669396	1.020022
_Ifoodcateg_14	.9705522	.0953434	-0.30	0.761	.7995839	1.178077
_Ifoodcateg_15	.9386221	.0376913	-1.58	0.116	.8671431	1.015993
_Ifoodcateg_16	.9524422	.0377781	-1.23	0.221	.8807643	1.029953
_Ifoodcateg_17	.9026801	.0488416	-1.89	0.060	.8113016	1.004351
_Ifoodcateg_18	.9414511	.0403535	-1.41	0.161	.8651248	1.024511
_Ifoodcateg_19	.8793147	.2125713	-0.53	0.595	.5458209	1.416571
_Ifoodcateg_20	.7898188	.0739947	-2.52	0.013	.6565546	.9501323
latitudepos	.99549	.0017427	-2.58	0.011	.9920583	.9989335
_Ioverallcu_2	.9232854	.1181992	-0.62	0.534	.7172431	1.188517
_Ioverallcu_3	.7762515	.1195734	-1.64	0.102	.572853	1.051869
_Ioverallcu_4	.9563678	.1209328	-0.35	0.725	.7452473	1.227296
_Ioverallcu_5	.8068878	.1248185	-1.39	0.167	.5946967	1.09479
_Ioverallcu_6	.6740234	.1178947	-2.26	0.025	.4773481	.951732
_Ioverallcu_8	.8918784	.0986276	-1.03	0.302	.7170886	1.109273
_Ioverallcu_9	.8342904	.0911658	-1.66	0.099	.6725232	1.034969
_Ioverallcu_10	.8264945	.1190835	-1.32	0.188	.6220287	1.09817
_Ioverallcu_11	.7174725	.0987231	-2.41	0.017	.5469283	.9411962
_Ipub_regio_2	1.005001	.0611766	0.08	0.935	.8912914	1.133217
_Ipub_regio_3	1.323236	.1530106	2.42	0.016	1.053365	1.662248
_Ipub_regio_4	1.054989	.0896776	0.63	0.530	.8921316	1.247575
_Ipub_regio_5	1.071205	.0796835	0.92	0.356	.9250141	1.240499
_Ipub_regio_6	1.079562	.1340172	0.62	0.538	.8450876	1.379093
_cons	1.573919	.1881803	3.79	0.000	1.243252	1.992535

In this very significant model comparator variables were ‘leafy green vegetables’, ‘Africa’, and ‘lack of culture method reported’. Iterative analyses controlling for confounders and interactions were conducted to determine the ranking of foods based on risk of carrying *Clostridium difficile* spores. Notice significance of latitude and total number of samples tested in the study. See manuscript for details.

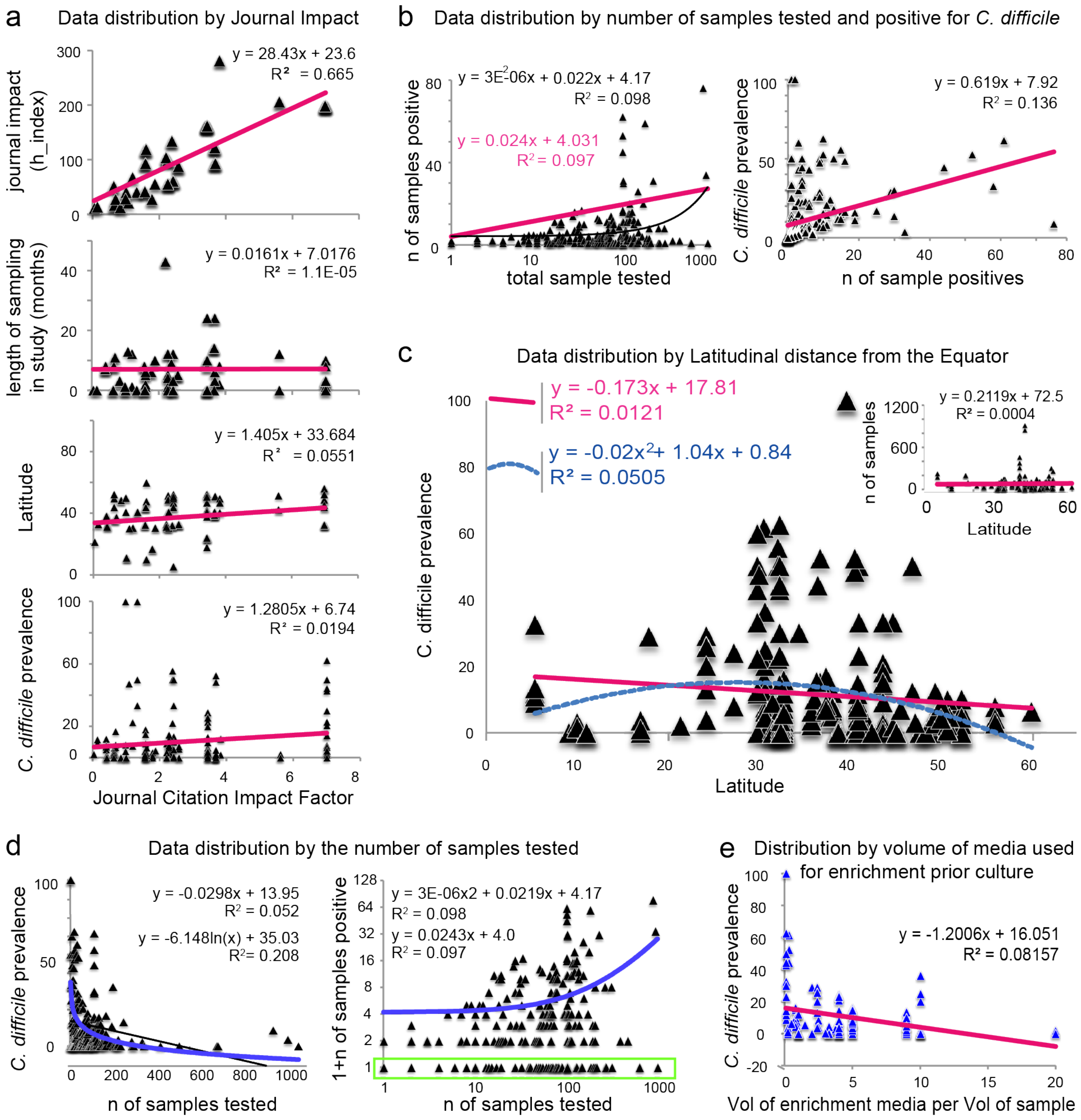

Supplementary Figure 1. Bivariate Scatterplots and Distribution of Prevalence Data as Function of Journal Quality, Latitude, Sample Size, and Volume of Liquid Media Used for Enrichment.

a) Scatter plots and linear correlations between the journal impact factor, and the variables 'length of the study, *C. difficile* prevalence, latitude, and the journal h-index, n=230 'food sample sets'. **b**) Scatterplot showing natural dependence between the number of samples tested and the number of positive samples, and prevalence. **c**) Scatter plot shows the linear and concave linear correlation as a function of latitude. Inset, notice the lack of direct correlation between latitude and the number of samples tested indicating that latitude has a consistent and direct effect on prevalence. **d**) Negative correlation between the prevalence and the number of samples tested, and dichotomy of studies publishing 'zero' prevalence for various food sample sets irrespective of the number of samples tested. **e**) The negative correlation between prevalence and the 'volume-of-liquid-media':'volume-of-sample' ratio (the slope) is influenced by an outlier report to the right.

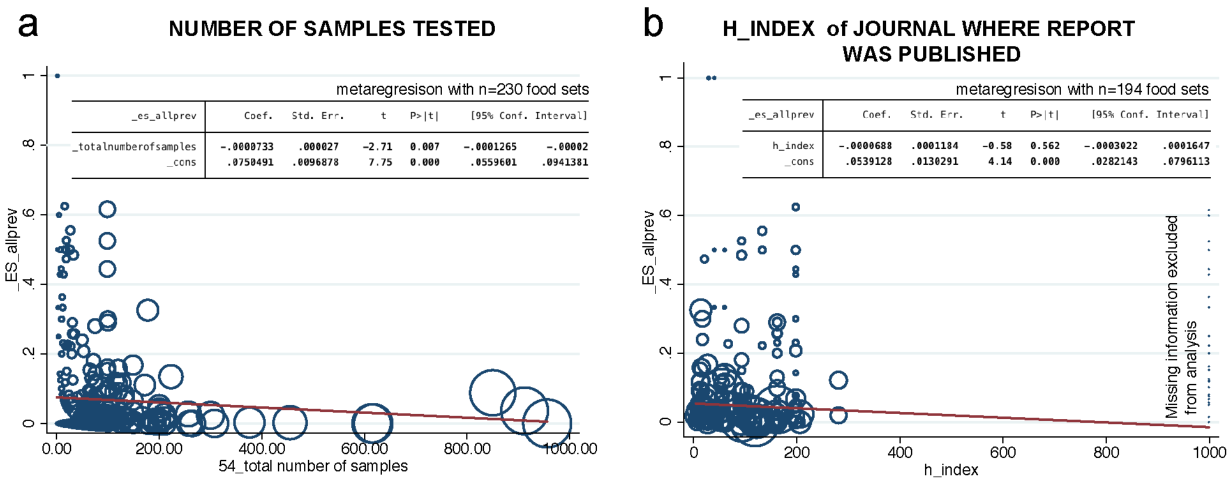

c

metaregression with n=170 food sets

```
Meta-regression                                    Number of obs  =      170
REML estimate of between-study variance            tau2           =        0
% residual variation due to heterogeneity          I-squared_res  =    0.00%
Proportion of between-study variance explained     Adj R-squared  =       .%
Joint test for all covariates                      Model F(3,166) =     1.15
With Knapp-Hartung modification                    Prob > F       =   0.3299
------------------------------------------------------------------------------
         _es_allprev |     exp(b)   Std. Err.      t    P>|t|     [95% Conf. Interval]
---------------------+--------------------------------------------------------
_totalnumberofsamples |    .999953   .0000255   -1.85   0.067     .9999026    1.000003
        impactfactor |   .9991944   .0082149   -0.10   0.922     .9831062    1.015546
             h_index |   1.000071   .0002054    0.34   0.732      .999665    1.000476
               _cons |   1.053815   .0203211    2.72   0.007     1.014448     1.09471
------------------------------------------------------------------------------
```

Supplementary Figure 2. Meta-regression analysis indicates that studies with large sample sizes tend to have inverse correlation with the *C. difficile* prevalence irrespective of journal quality.

a) Significant meta-regression linear estimate and correlation predicted coefficients around weighted prevalence estimates (based on FT-double arcsine transformation of reported estimates) for *C. difficile* vs. the number of food samples tested across all studies (n=230 food sets). Meta-regression adjusted P=0.007. **b**) Nonsignificant meta-regression estimates and correlation prediction around weighted estimates for H-index of journal where studies were published, (n=194 food sets). Meta-regression adjusted P=0.5. **c**) Multivariable meta-regression coefficients show interaction effects with journal quality, lack of effect for h-index and impact factor, but a significant negative trend (exp(b) estimate <1.0) for the total number of samples tested in each study (adjusted P=0.067). Studies with larger samples sizes are more likely to report lower CD prevalence.

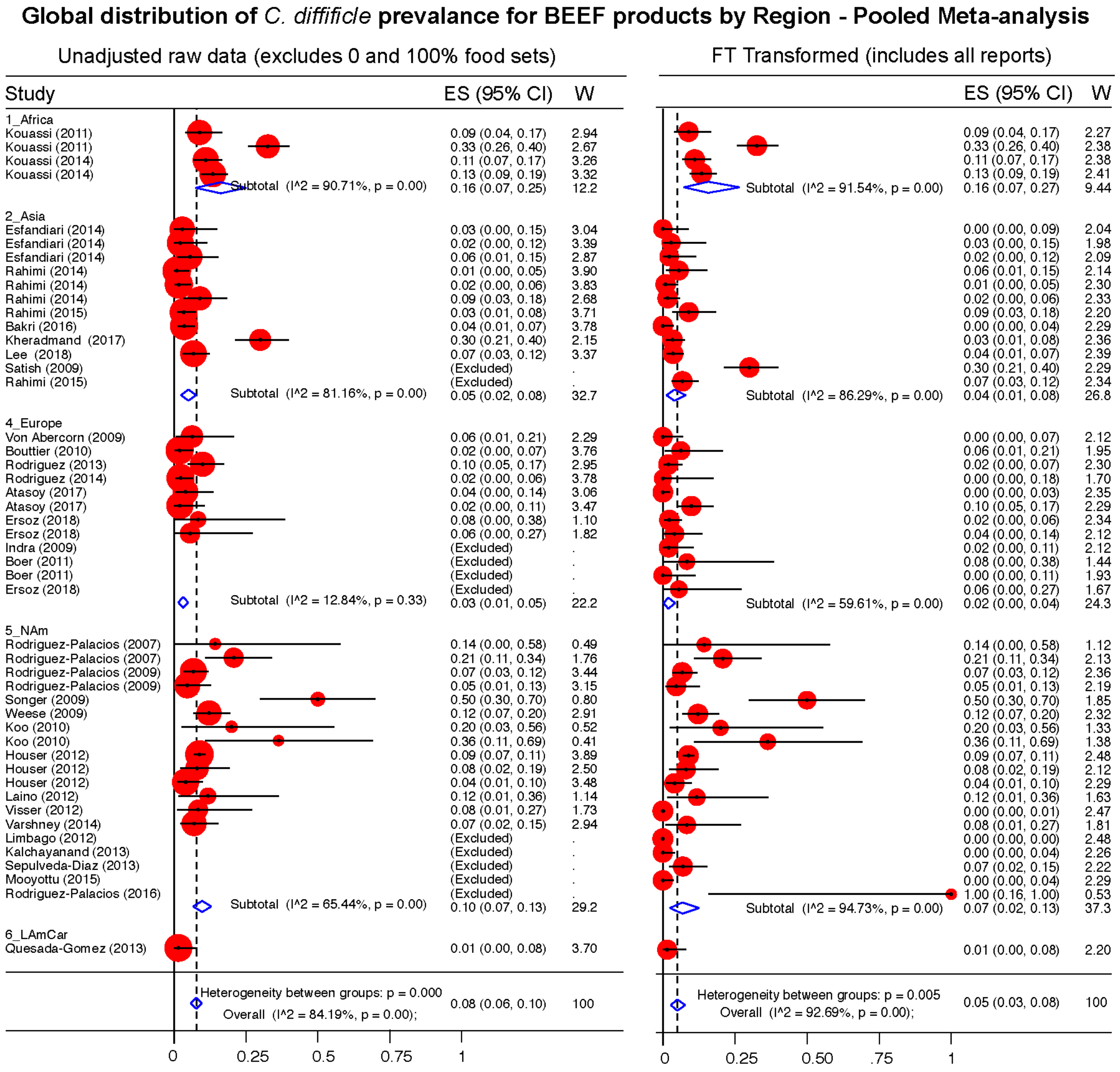

Supplementary Figure 3. Beef - Global and Regional Prevalence of *C. difficile* in products intended for human consumption. Weighed Meta-analysis Estimates and Forest Plot across regions using both untransformed reported prevalence data (left panel) and Freeman-Tukey Double Arcsine Transformation (right panel).

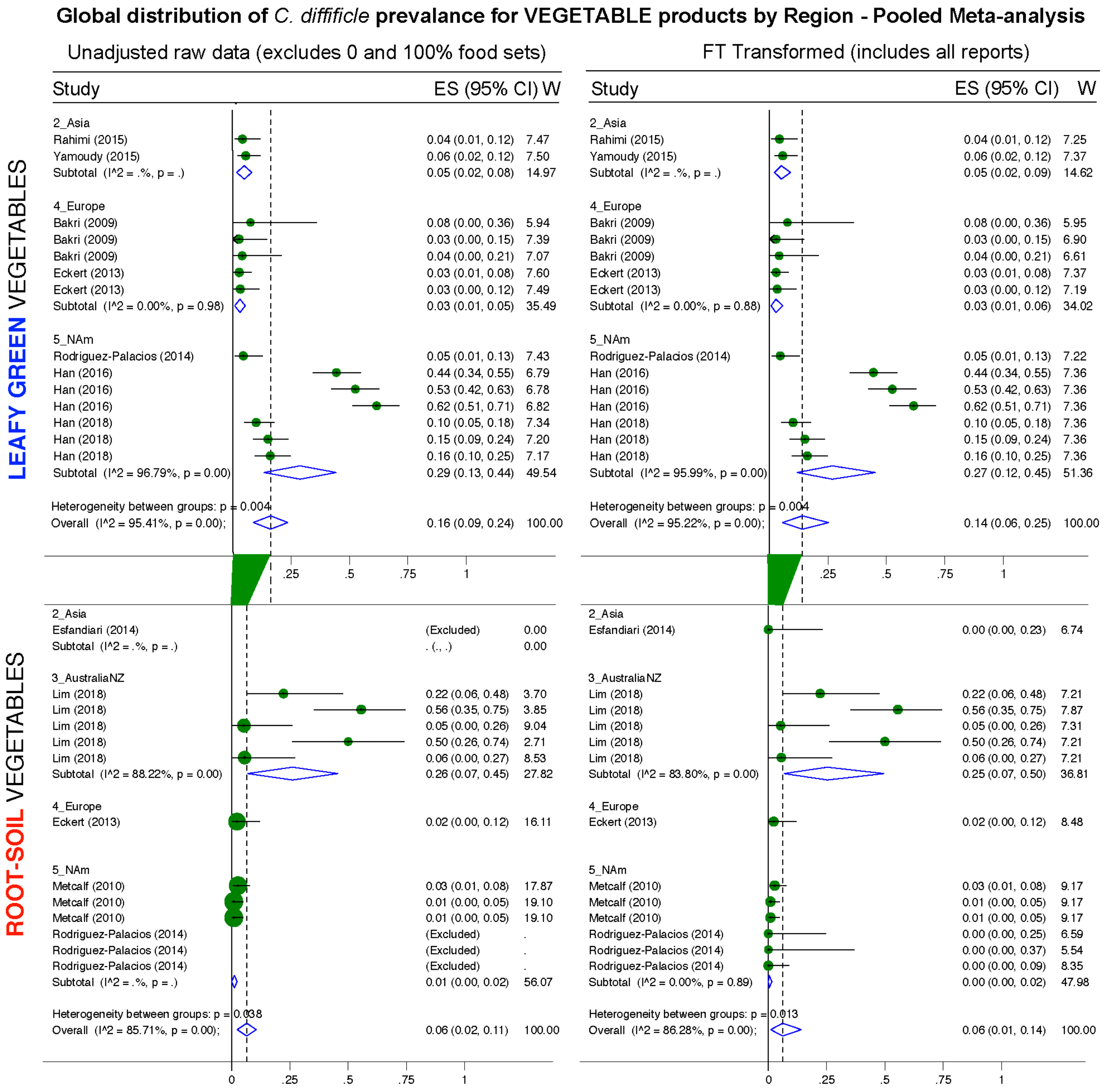

Supplementary Figure 4. Leafy Green and Root/soil Vegetables - Global and Regional Prevalence of *C. difficile* in products intended for human consumption. Weighed Meta-analysis Estimates and Forest Plot across regions using both untransformed reported prevalence data (left panel) and Freeman-Tukey Double Arcsine Transformation (right panel). Notice that the difference between the overall estimates for the leafy vegetables and the root vegetables is similar independently of data adjustment methods used (see green polygon in between the top and bottom panels).

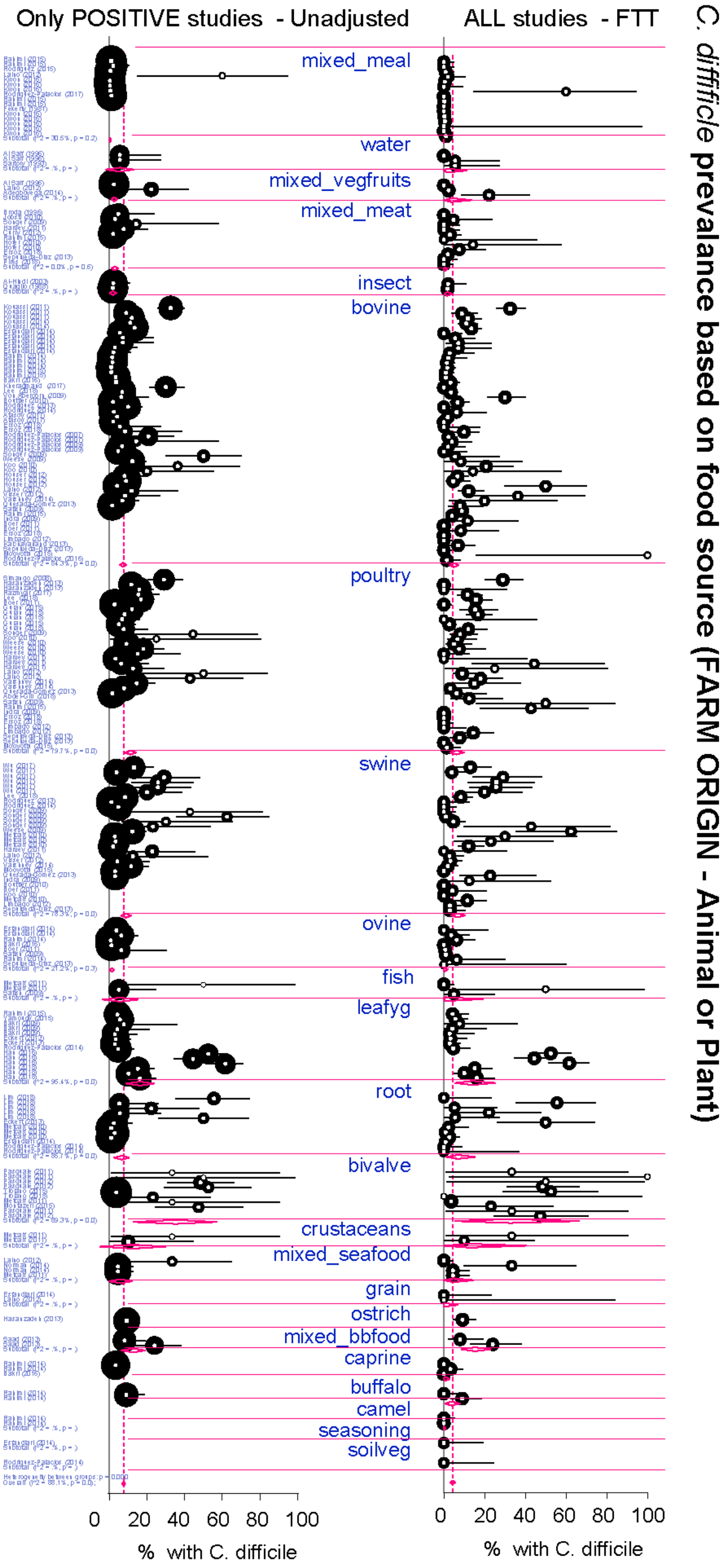

Supplementary Figure 5. Pooled meta-analysis of food categories based on origin of food (animal vs plant).

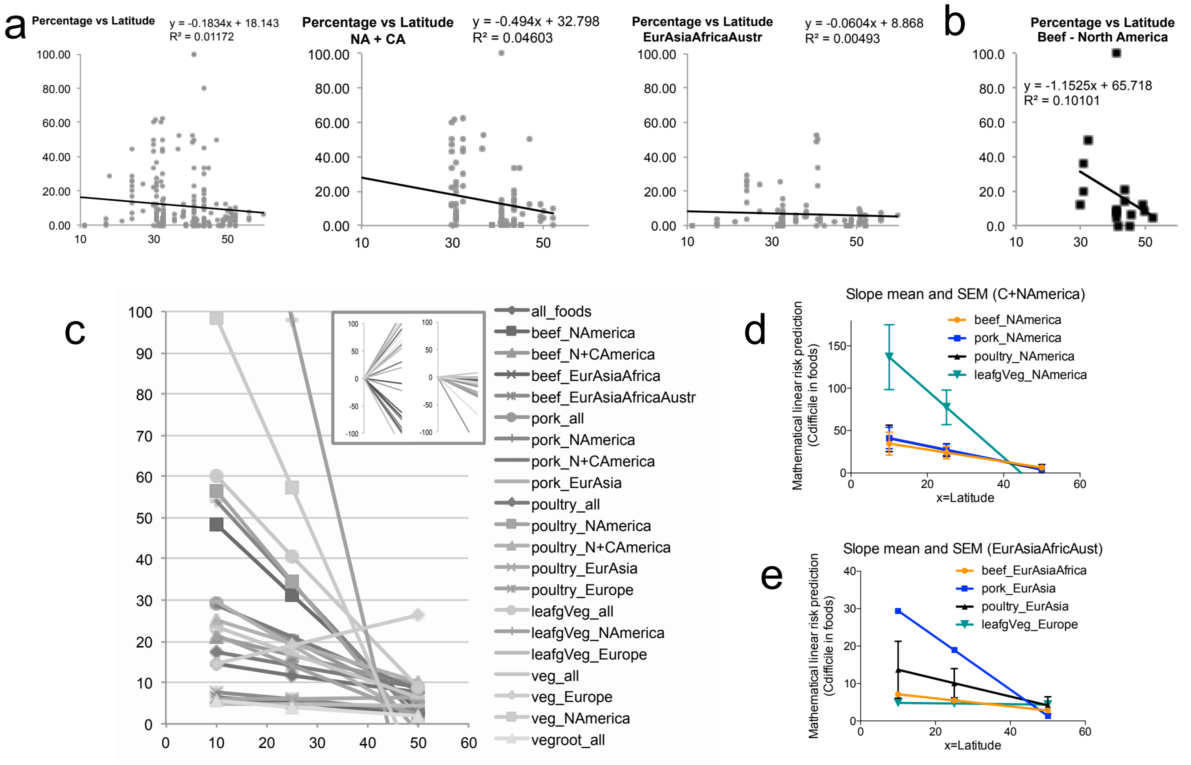

Supplementary Figure 6. The negative correlation between the presence of *C. difficile* in foods and the study latitude occurs across several regions and food categories.

a) Scatter plot of raw extracted percentage data from studies and latitude, for all samples, and for the Americas (NA+CA) and the rest of the world (EurAsiaAfricaAustr). Notice the linear trend and the negative value of slope (e.g, y=-0.1834x in left panel) **b**) Scatter plot of raw extracted percentage data from studies and latitude, for only beef studies in North America. **c**) Scatter plot of simulated data computed using linear trend equations determined for raw extracted percentage data from studies and latitude, across various region-by-food combinations. **d**) Summary plot using mean slopes to compute the trends for four major food categories (Beef, Poultry, Pork and Leafy green vegetables) for the Americas and the rest of the world, separately.

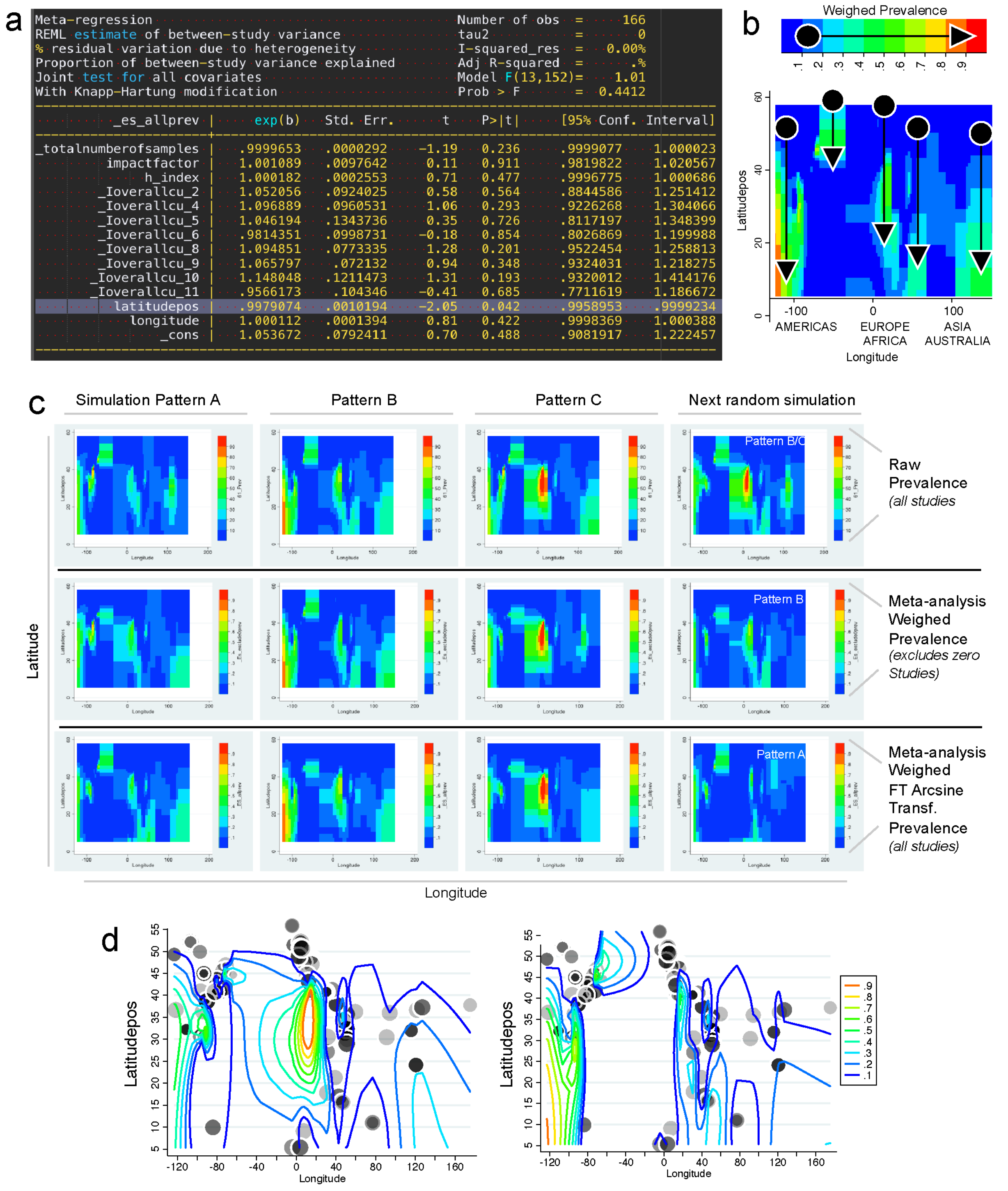

```
Meta-regression                                      Number of obs  =     166
REML estimate of between-study variance              tau2           =       0
% residual variation due to heterogeneity            I-squared_res  =   0.00%
Proportion of between-study variance explained       Adj R-squared  =      .%
Joint test for all covariates                        Model F(13,152)=    1.01
With Knapp-Hartung modification                      Prob > F       =  0.4412
```

_es_allprev	exp(b)	Std. Err.	t	P>|t|	[95% Conf. Interval]	
_totalnumberofsamples	.9999653	.0000292	-1.19	0.236	.9999077	1.000023
impactfactor	1.001089	.0097642	0.11	0.911	.9819822	1.020567
h_index	1.000182	.0002553	0.71	0.477	.9996775	1.000686
_Ioverallcu_2	1.052056	.0924025	0.58	0.564	.8844586	1.251412
_Ioverallcu_4	1.096889	.0960531	1.06	0.293	.9226268	1.304066
_Ioverallcu_5	1.046194	.1343736	0.35	0.726	.8117197	1.348399
_Ioverallcu_6	.9814351	.0998731	-0.18	0.854	.8026869	1.199988
_Ioverallcu_8	1.094851	.0773335	1.28	0.201	.9522454	1.258813
_Ioverallcu_9	1.065797	.072132	0.94	0.348	.9324031	1.218275
_Ioverallcu_10	1.148048	.1211473	1.31	0.193	.9320012	1.414176
_Ioverallcu_11	.9566173	.104346	-0.41	0.685	.7711619	1.186672
latitudepos	.9979074	.0010194	-2.05	0.042	.9958953	.9999234
longitude	1.000112	.0001394	0.81	0.422	.9998369	1.000388
_cons	1.053672	.0792411	0.70	0.488	.9081917	1.222457

Supplementary Figure 7. Meta-regression and simulations indicate that the effect of latitude on the prevalence of *C. difficile* is reproducible and independent of data adjustment and culture approaches. a) Multivariable meta-regression demonstrate that the significant effect of latitude is independent of culture method (_Ioverallcu_). **b**) Random simulation contour density plot depicts CD prevalence trends over a 2D-latitude/longitude plane. **c**) Contour plot simulation patterns show that the latitudinal trends highlighted in panel b are reproducible regardless of the data adjustment approach. d) Contour line plot simulations depict the study location and weighed meta-regression contribution to the analysis.

Supplementary Methods

Risk of Bias. Risk of bias was assessed for all studies by rating each of the 6 quality assessment items listed in the DET into dichotomous ratings: low risk (1) and high risk (0). An overall Risk of Bias score was calculated by adding the numeric value of all six items. High scores indicate low risk of bias and stronger method quality. The only quality assessment question used as exclusion criteria for data extraction was failure to report raw/unadjusted or adjusted data on prevalence. Measures of data SD or variability were estimated using the number of food samples tested and the percentage of positive samples. Because the reliability of available statistical methods on bias have previously shown to be inaccurate and misleading with effects that are close to the extremes, for instance close to 0 or to 100% [16], publication bias was tested using funnel plot and Egger's statistics using study size at the food set level instead of the standard error of the effect as recommended for proportions with high data /effects polarity [16]. As we recently mentioned [17] however, it is uncertain how many studies start but do not get published due to the lack of a prepublication registry of prevalence based studies in foods. Studies reporting contamination of early stage food products (animal carcasses)[46] and foods destined for companion animals were excluded [47, 48].

Pooled Ratios and Meta-analysis Extracted data were used to estimate risk ratios and perform a prevalence meta-analysis. Three main categories of data were extracted: characteristics of the samples, methodological characteristics of each study, and prevalence data. The primary outcome of the study was prevalence of CD in foods at any point of food production chain. Prevalence rates were calculated from raw proportions or as percentages calculated from raw denominator (positive samples reported) and numerator (total number of samples) reported in the selected studies. Root and vegetable products that grow on the soil surface (e.g., cucurbids), were pooled as one commodity designated root/soil vegetables. Leafy greens and mixed vegetables and fruits were analyzed separately. Mixed vegetables were unspecified species of fruits and vegetables. Meat products include hamburgers and sausages. Fish, crustaceans and bivalves were pooled as Seafood.

We calculated pooled risk ratios (RR, 95% CI) for each food group to quantify the differences and rank the foods according to the risk of being contaminated with CD using a random effects model [18] as we previously reported.[19] In brief, heterogeneity tests with Higgins' I^2 statistic were performed to determine the extent of variation between the studies that rely on measure analysis for the deviations for each within-study variance from a central estimate for the collective between-study variance distribution. [18] I2 describes the percentage of total variation across-studies that is due to heterogeneity rather than chance (0% indicates no heterogeneity, >0 indicates heterogeneity). To quantify the inconsistencies across studies we interpreted I^2 statistics following guidelines proposed by Higgins [49], in which detectable inconsistencies do not necessarily affect the conclusion of the overall estimates. Meta-analysis was used to estimate the overall prevalence of CD in foods globally and per region by pooling variances of proportions or percentages in a random-effects model using DerSimonian and Laird method. [20, 21] We calculated the pooled estimates and the 95% CIs in studies and considered non-overlapping CIs as an indication of statistically significant differences. Freeman-Turkey transformation and Miller's inverse were used to transform the zero prevalence observations, [50, 51] although logit based statistics are also possible to control for variability as an alternative to arcsine transformation. [52] Meta-analyses were performed on transformed data using R software and the package *Metaphor* [22], of Stata software and available meta-analysis and meta-regression functions. Exact binomial weighted and pooled estimates are presented in forest plots without adjusting for 'zero-studies' (which excludes 0% prevalence studies), and with adjustments using a balanced addition of 1 unit to the n and N, and using the Freeman-Tukey double arcsine transformation, both methods to include 0% prevalence studies as recommended. [22]

Meta-regression. Latitude and longitude data was obtained from the latitude/longitude finder database from NASA (https://mynasadata.larc.nasa.gov/latitudelongitude-finder/). For studies not reporting city data to infer the latitudinal data, we assigned latitude data based on the city locality where testing occurred. Studies from nationwide network surveillance systems across the USA were excluded from this meta-regression given the lack of sample origin information. [53, 54] To determine if the reported prevalences were influenced by the amount of food tested data, when available data were extracted as absolute values in grams (with exceptions for normalization for volumetric units which were converted to grams at a 1:1 v:g ratio; surface rinsate volumes were approximated to 0.1g per 10 ml of rinsate; and surface areas sampled with sponges or swabs were approximated to 0.2 grams; the food weight/surface area to rinsate ratio variance was assumed to be randomly distributed across samples). Modeling was conducted following principles and using strategies described in documentation packages in R and STATA. [22]

Supplementary References

1. Fekety R, Kim KH, Brown D, et al. Epidemiology of antibiotic-associated colitis; isolation of Clostridium difficile from the hospital environment. Am J Med 1981;70:906-8.
2. Rodriguez-Palacios A, Staempfli HR, Duffield T, et al. Clostridium difficile in retail ground meat, Canada. Emerg Infect Dis 2007;13:485-7.
3. Rodriguez-Palacios A, Reid-Smith RJ, Staempfli HR, et al. Possible seasonality of Clostridium difficile in retail meat, Canada. Emerg Infect Dis 2009;15:802-5.
4. Songer JG, Trinh HT, Killgore GE, et al. Clostridium difficile in retail meat products, USA, 2007. Emerg Infect Dis 2009;15:819-21.
5. Weese JS, Avery BP, Rousseau J, et al. Detection and enumeration of Clostridium difficile spores in retail beef and pork. Appl Environ Microbiol 2009;75:5009-11.
6. Metcalf DS, Costa MC, Dew WM, et al. *Clostridium difficile* in vegetables, Canada. Lett Appl Microbiol 2010;51:600-602.
7. Metcalf D, Reid-Smith RJ, Avery BP, et al. Prevalence of Clostridium difficile in retail pork. Can Vet J 2010;51:873-6.
8. Weese JS, Reid-Smith RJ, Avery BP, et al. Detection and characterization of Clostridium difficile in retail chicken. Lett Appl Microbiol 2010;50:362-5.
9. Koo H, Darkoh C, Koo DC, et al. Potential Foodborne Transmission of *Clostridium Difficile* Infection In a Hospital Setting. IDSA press conferences. Volume 3-2-2013, 2010.
10. Harvey RB, Norman KN, Andrews K, et al. Clostridium difficile in retail meat and processing plants in Texas. J Vet Diagn Invest 2011;23:807-11.
11. Harvey RB, Norman KN, Andrews K, et al. Clostridium difficile in poultry and poultry meat. Foodborne Pathog Dis 2011;8:1321-3.
12. Metcalf D, Avery BP, Janecko N, et al. Clostridium difficile in seafood and fish. Anaerobe 2011;17:85-6.
13. Visser M, Sephri S, Sepehrim S, et al. Detection of Clostridium difficile in retail ground meat products in Manitoba. Can J Infect Dis Med Microbiol 2012;23:28-30.
14. Limbago B, Thompson AD, Greene SA, et al. Development of a consensus method for culture of Clostridium difficile from meat and its use in a survey of U.S. retail meats. Food Microbiol 2012;32:448-51.
15. Curry SR, Marsh JW, Schlackman JL, et al. Prevalence of Clostridium difficile in uncooked ground meat products from Pittsburgh, Pennsylvania. Appl Environ Microbiol 2012;78:4183-6.
16. Houser BA, Soehnlen MK, Wolfgang DR, et al. Prevalence of Clostridium difficile toxin genes in the feces of veal calves and incidence of ground veal contamination. Foodborne Pathog Dis 2012;9:32-6.
17. Laino C, Martin LJ. Hospital food contaminated with C diff. Volume 3-2-2013: WebMD Health News, 2012.
18. Sepulveda Diaz RV. Prevalence of Clostridium difficile in retail meats from Minnesota and comparison of growth and survival of human and animal isolates. Retrieved from the University of Minnesota Digital Conservancy, http://hdl.handle.net/11299/160297., 2013.
19. Shaughnessy MK, Snider T, Sepulveda R, et al. Prevalence and Molecular Characteristics of Clostridium difficile in Retail Meats, Food-Producing and Companion Animals, and Humans in Minnesota. J Food Prot 2018;81:1635-1642.
20. Kalchayanand N, Arthur TM, Bosilevac JM, et al. Isolation and characterization of Clostridium difficile associated with beef cattle and commercially produced ground beef. J Food Prot 2013;76:256-64.
21. Norman KN, Harvey RB, Andrews K, et al. Survey of Clostridium difficile in retail seafood in College Station, Texas. Food Addit Contam Part A Chem Anal Control Expo Risk Assess 2014;31:1127-9.
22. Rodriguez-Palacios A, Ilic S, LeJeune JT. Clostridium difficile with Moxifloxacin/Clindamycin Resistance in Vegetables in Ohio, USA, and Prevalence Meta-Analysis. J Pathog 2014;2014:158601.
23. Varshney JB, Very KJ, Williams JL, et al. Characterization of Clostridium difficile isolates from human fecal samples and retail meat from Pennsylvania. Foodborne Pathog Dis 2014;11:822-9.
24. Montazeri N, Liu D, Janes ME. Occurrence of Toxigenic *Clostridium difficile* in Louisiana Oysters (*Crassostrea virginica*) and Environmental Waters. Food and Nutrition Sciences 2015;6:1065-1070.
25. Mooyottu S, Flock G, Kollanoor-Johny A, et al. Characterization of a multidrug resistant C. difficile meat isolate. Int J Food Microbiol 2015;192:111-6.
26. Kwon JH, Lanzas C, Reske KA, et al. An Evaluation of Food as a Potential Source for Clostridium difficile Acquisition in Hospitalized Patients. Infect Control Hosp Epidemiol 2016:1-7.
27. Rodriguez-Palacios A, Ilic S, LeJeune JT. Subboiling Moist Heat Favors the Selection of Enteric Pathogen Clostridium difficile PCR Ribotype 078 Spores in Food. Can J Infect Dis Med Microbiol 2016;2016:1462405.
28. Han Y. Detection of Antibiotic Resistance Clostridium difficile in Lettuce. MSc Thesis. Louisiana State University. https://digitalcommons.lsu.edu/gradschool_theses/1516, 2016.
29. Han Y, King J, Janes ME. Detection of antibiotic resistance toxigenic *Clostridium difficile* in processed retail lettuce. Food Quality and Safety 2018;00:1.
30. Rodriguez-Palacios A, Ilic S, LeJeune JT. Food Indwelling Clostridium difficile in Naturally Contaminated Household Meals: Data for Expanded Risk Mathematical Predictions. Infect Control Hosp Epidemiol 2017;38:509-510.
31. Quaglio P, Messi P, Fabio A. Bacterial isolates of the genus Clostridium in honey samples. Igiene Moderna 1988;90:486-496.
32. Sartory DP, Pritchard AM, Holmes P. ENUMERATION OF SULFITE-REDUCING CLOSTRIDIA FROM POTABLE WATER-SUPPLIES. Water Science and Technology 1993;27:279-282.
33. al Saif N, Brazier JS. The distribution of Clostridium difficile in the environment of South Wales. J Med Microbiol 1996;45:133-7.
34. Indra A, Lassnig H, Baliko N, et al. Clostridium difficile: a new zoonotic agent? Wien Klin Wochenschr 2009;121:91-5.
35. Abercron SMMV, Wierup M, Krovacek K, et al. Low Occurrence of Clostridium difficile in Retail Ground Meat in Sweden. Journal of food protection 2009;72:1732-1734.

36. Karlsson F. Finns Clostridium difficile i köttprodukter i Sverige? Dept. of Biomedical Sciences and Veterinary Public Health, Uppsala. Uppsala: SLU, Dept. of Biomedical Sciences and Veterinary Public Health, 2009.
37. Bakri MM, Brown DJ, Butcher JP, et al. Clostridium difficile in ready-to-eat salads, Scotland. Emerg Infect Dis 2009;15:817-8.
38. Bouttier S, Barc MC, Felix B, et al. Clostridium difficile in ground meat, France. Emerg Infect Dis 2010;16:733-5.
39. Jobstl M, Heuberger S, Indra A, et al. Clostridium difficile in raw products of animal origin. Int J Food Microbiol 2010.
40. Hoffer E, Haechler H, Frei R, et al. Low occurrence of Clostridium difficile in fecal samples of healthy calves and pigs at slaughter and in minced meat in Switzerland. J Food Prot 2010;73:973-5.
41. de Boer E, Zwartkruis-Nahuis A, Heuvelink AE, et al. Prevalence of Clostridium difficile in retailed meat in the Netherlands. Int J Food Microbiol 2011;144:561-4.
42. Pasquale V, Romano VJ, Rupnik M, et al. Isolation and characterization of Clostridium difficile from shellfish and marine environments. Folia Microbiol (Praha) 2011;56:431-7.
43. Pasquale V, Romano V, Rupnik M, et al. Occurrence of toxigenic Clostridium difficile in edible bivalve molluscs. Food Microbiol 2012;31:309-12.
44. Eckert C, Burghoffer B, Barbut F. Contamination of ready-to-eat raw vegetables with Clostridium difficile in France. J Med Microbiol 2013;62:1435-8.
45. Rodriguez C, Avesani V, Van Broeck J, et al. Presence of Clostridium difficile in pigs and cattle intestinal contents and carcass contamination at the slaughterhouse in Belgium. Int J Food Microbiol 2013;166:256-62.
46. Rodriguez C, Taminiau B, Avesani V, et al. Multilocus sequence typing analysis and antibiotic resistance of Clostridium difficile strains isolated from retail meat and humans in Belgium. Food Microbiol 2014;42:166-71.
47. Rodriguez C, Korsak N, Taminiau B, et al. Clostridium difficile from food and surface samples in a Belgian nursing home: An unlikely source of contamination. Anaerobe 2015;32:87-9.
48. Troiano T, Harmanus C, Sanders IM, et al. Toxigenic Clostridium difficile PCR ribotypes in edible marine bivalve molluscs in Italy. Int J Food Microbiol 2015;208:30-4.
49. Guran HS, Ilhak OI. Clostridium difficile in retail chicken meat parts and liver in the Eastern Region of Turkey. Journal für Verbraucherschutz und Lebensmittelsicherheit 2015;10:359-364.
50. Atasoy F, Gucukoglu A. Detection of *Clostridium difficile* and toxin genes in samples of modified atmosphere packaged (MAP) minced and cubed beef meat. Ankara Üniv Vet Fak Derg 2017;64:165-170.
51. Ersöz Ş, Coşansu S. Prevalence of *Clostridium difficile* Isolated from Beef and Chicken Meat Products in Turkey. Korean J Food Sci Anim Resour 2018;38:759-767.
52. Primavilla S, Farneti S, Petruzzelli A, et al. Contamination of hospital food with Clostridium difficile in Central Italy. Anaerobe 2018;55:8-10.
53. Tkalec V, Janezic S, Skok B, et al. High Clostridium difficile contamination rates of domestic and imported potatoes compared to some other vegetables in Slovenia. Food Microbiol 2019;78:194-200.
54. Al-Hindi RR. Microbiological qualities of imported and locally produced honey in Saudi Arabia. Arab Gulf Journal of Scientific Research 2003;21:204-209.
55. Sathish S, Swaminathan K. Genetic diversity among toxigenic clostridia isolated from soil, water, meat and associated polluted sites in South India. Indian Journal of Medical Microbiology 2009;27:311-320.
56. Hasanzadeh A, Rahimi E. Isolation of Clostridium difficile from chicken meat sold in meat stores of Isfahan City. Advances in Environmental Biology 2013;7:2372-2374.
57. Hasanzade A, Rahimi E. Isolation of Clostridium difficile from turkey and ostrich meat sold in meat stores of Isfahan City. International Journal of Advanced Biological and Biomedical Research 2013;1:963-967.
58. Rahimi E, Jalali M, Weese JS. Prevalence of Clostridium difficile in raw beef, cow, sheep, goat, camel and buffalo meat in Iran. BMC Public Health 2014;14:119.
59. Rahimi E, Momtaz H, Hemmati M. Occurrence of Clostridium difficile in Raw Bovine, Ovine, Caprine, Camel and Buffalo Milk in Iran. Kafkas Universitesi Veteriner Fakultesi Dergisi 2014;20:371-374.
60. Esfandiari Z, Weese S, Ezzatpanah H, et al. Occurrence of Clostridium difficile in seasoned hamburgers and seven processing plants in Iran. BMC Microbiol 2014;14:283.
61. Esfandiari Z, Jalali M, Ezzatpanah H, et al. Prevalence and Characterization of Clostridium difficile in Beef and Mutton Meats of Isfahan Region, Iran. Jundishapur J Microbiol 2014;7:e16771.
62. Yamoudy M, Mirlohi M, Isfahani BN, et al. Isolation of toxigenic Clostridium difficile from ready-to-eat salads by multiplex polymerase chain reaction in Isfahan, Iran. Adv Biomed Res 2015;4:87.
63. Rahimi E, Khaksar F. Detection of toxigenic *Clostridium difficile* strains isolated from meat and meat products in Iran. Bulgarian Journal of Veterinary Medicine 2015;18:277-281.
64. Rahimi E, Afzali ZS, Baghbadorani ZT. *Clostridium difficile* in ready-to-eat foods in Isfahan and Shahrekord, Iran. Asian Pacific Journal of Tropical Biomedicine 2015;5:128-131.
65. Bakri M. Prevalence of Clostridium difficile in raw cow, sheep, and goat meat in Jazan, Saudi Arabia. Saudi Journal of Biological Sciences 2016.
66. Wu YC, Chen CM, Kuo CJ, et al. Prevalence and molecular characterization of Clostridium difficile isolates from a pig slaughterhouse, pork, and humans in Taiwan. Int J Food Microbiol 2017;242:37-44.
67. Kheradmand M, Jalilian S, Alvandi A, et al. Prevalence of Clostridium difficile and its toxigenic genotype in beef samples in west of Iran. Iran J Microbiol 2017;9:169-173.
68. Razmyar J, Jamshidi A, Khanzadi S, et al. Toxigenic Clostridium difficile in retail packed chicken meat and broiler flocks in northeastern Iran. Iran J Vet Res 2017;18:271-274.
69. Lee JY, Lee DY, Cho YS. Prevalence of Clostridium difficile isolated from various raw meats in Korea. Food Science and Biotechnology 2018:1-7.
70. Nayebpour F, Rahimi E. Prevalence, antibiotic resistance, and toxigenic gene profile of the *Clostridium difficile* isolated from molluscan shellfish. Journal of Food Safety 2018;e12586, https://doi.org/10.1111/jfs.12586.
71. Simango C, Mwakurudza S. *Clostridium difficile* in broiler chickens sold at market places in Zimbabwe and their antimicrobial susceptibility. Int J Food Microbiol 2008;124:268-70.

72. Kouassi KA, Dadie AT, Nanga ZY, et al. Prevalence of sulfite reducing Clostridium species in barbecued meat in Abidjan, Côte d'Ivoire. Journal of Applied Biosciences 2011;38:2518-2522.
73. Saad NM, Amin WF, Shaker EM. Detection of toxigenic Clostridium difficile in powdered infant and follow-up formulae in Egypt. Veterinary World 2013;6:862-864.
74. Kouassi KA, Dadie AT, N'Guessan KF, et al. Clostridium perfringens and Clostridium difficile in cooked beef sold in Côte d'Ivoire and their antimicrobial susceptibility. Anaerobe 2014;28:90-4.
75. Adegboyega T. Environmental Sources *Clostridium difficile* in Lagos State, Nigeria. Journal of Health, Medicine and Nursing 2014;3.
76. Abdel-Glil MY, Thomas P, Schmoock G, et al. Presence of Clostridium difficile in poultry and poultry meat in Egypt. Anaerobe 2018;51:21-25.
77. Broda DM, DeLacy KM, Bell RG, et al. Psychrotrophic Clostridium spp. associated with 'blown pack' spoilage of chilled vacuum-packed red meats and dog rolls in gas-impermeable plastic casings. Int J Food Microbiol 1996;29:335-52.
78. Lim SC, Foster NF, Elliott B, et al. High prevalence of Clostridium difficile on retail root vegetables, Western Australia. J Appl Microbiol 2017.
79. Quesada-Gómez C, Mulvey MR, Vargas P, et al. Isolation of a toxigenic and clinical genotype of clostridium difficile in retail meats in Costa Rica. J Food Prot 2013;76:348-51.
80. Pires RN, Caurio CFB, Saldanha GZ, et al. Clostridium difficile contamination in retail meat products in Brazil. Braz J Infect Dis 2018;22:345-346.
81. Norman KN, Andrews K, Brawley AD, et al. Varied prevalence of Clostridium difficile in an integrated swine operation [electronic resource]. Anaerobe 2009;15:256-260.
82. Rodriguez-Palacios A, LeJeune JT, Loerch S, et al. Transient Fecal Shedding and Limited Animal-to-Animal Transmission of Clostridium difficile by Naturally Infected Finishing Feedlot Cattle. Applied and environmental microbiology AEM 2011;77:3391-3397.
83. Hawken P, Weese JS, Friendship R, et al. Longitudinal study of Clostridium difficile and Methicillin-resistant Staphylococcus aureus associated with pigs from weaning through to the end of processing. J Food Prot 2013;76:624-30.
84. Hawken P, Scott Weese J, Friendship R, et al. Carriage and dissemination of Clostridium difficile and methicillin resistant Staphylococcus aureus in pork processing. Food Control 2013;31:433-437.
85. Knight DR, Putsathit P, Elliott B, et al. Contamination of Australian newborn calf carcasses at slaughter with Clostridium difficile. Clin Microbiol Infect 2016;22:266.e1-7.
86. Hunter JP, Saratzis A, Sutton AJ, et al. In meta-analyses of proportion studies, funnel plots were found to be an inaccurate method of assessing publication bias. J Clin Epidemiol 2014;67:897-903.
